# Natural antisense RNA Foxk1-AS promotes myogenic differentiation by inhibiting Foxk1 activity

**DOI:** 10.1186/s12964-022-00896-2

**Published:** 2022-05-31

**Authors:** Chun Li, Hao Shen, Meng Liu, Siguang Li, Yuping Luo

**Affiliations:** 1 Clinical and Translational Research Center, Shanghai First Maternity and Infant Hospital, Tongji University School of Medicine, Shanghai, 201204 People’s Republic of China; 2 Key Laboratory of Spine and Spinal Cord Injury Repair and Regeneration of Ministry of Education, Orthopedic Department of Tongji Hospital, Tongji University School of Medicine, Shanghai, 200065 People’s Republic of China; 3 School of Life Science, Nanchang University, Nanchang, 330031 People’s Republic of China

**Keywords:** Natural antisense RNA, *Foxk1-AS*, *Foxk1*, Myogenic differentiation, *Mef2c*

## Abstract

**Background:**

Natural antisense RNAs are RNA molecules that are transcribed from the opposite strand of either protein-coding or non-protein coding genes and have the ability to regulate the expression of their sense gene or several related genes. However, the roles of natural antisense RNAs in the maintenance and myogenesis of muscle stem cells remain largely unexamined.

**Methods:**

We analysed myoblast differentiation and regeneration by overexpression and knockdown of *Foxk1*-*AS* using lentivirus and adeno-associated virus infection in C2C12 cells and damaged muscle tissues. Muscle injury was induced by BaCl_2_ and the regeneration and repair of damaged muscle tissues was assessed by haematoxylin–eosin staining and quantitative real-time PCR. The expression of myogenic differentiation-related genes was verified via quantitative real-time PCR, Western blotting and immunofluorescence staining.

**Results:**

We identified a novel natural antisense RNA, *Foxk1*-*AS*, which is transcribed from the opposite strand of *Foxk1* DNA and completely incorporated in the 3′ UTR of *Foxk1*. *Foxk1*-*AS* targets *Foxk1* and functions as a regulator of myogenesis. Overexpression of *Foxk1*-*AS* strongly inhibited the expression of *Foxk1* in C2C12 cells and in tibialis anterior muscle tissue and promoted myoblast differentiation and the regeneration of muscle fibres damaged by BaCl_2_. Furthermore, overexpression of *Foxk1*-*AS* promoted the expression of *Mef2c*, which is an important transcription factor in the control of muscle gene expression and is negatively regulated by *Foxk1*.

**Conclusion:**

The results indicated that *Foxk1*-*AS* represses *Foxk1*, thereby rescuing Mef2c activity and promoting myogenic differentiation of C2C12 cells and regeneration of damaged muscle fibres.

**Video Abstract**

**Supplementary Information:**

The online version contains supplementary material available at 10.1186/s12964-022-00896-2.

## Background

Myogenesis in adults is a highly regulated process that includes activation of muscle stem cells (MuSCs), followed by proliferation and differentiation of myoblasts and subsequent fusion of myoblasts to produce multinucleated myotubes, which further mature into myofibres [1]. Several factors precisely regulate the differentiation stage of skeletal muscle cells [1–4]. In addition to myogenic regulatory factors (MRFs), including myogenic differentiation 1 (MyoD), myogenic factor 5 (Myf5), myogenin (MyoG) and myogenic regulatory factor 4 (Mrf4), the transcription factor forkhead box K 1 (Foxk1) is involved in muscle cell differentiation and skeletal muscle regeneration [5–8]. The Foxk1 protein acts as a transcriptional regulator and inhibits myogenic differentiation by repressing myocyte enhancer factor 2 (Mef2) activity [7]. Despite these recent insights, the regulatory elements that govern Foxk1 activation and then regulate myogenesis are not entirely understood.

Long noncoding RNAs (lncRNAs) are involved in numerous important biological processes [9, 10]. Recent research has shown that lncRNAs play an important role in regulating myogenesis and skeletal muscle regeneration by interacting with various proteins or acting as molecular sponges for miRNAs. For instance, MUNC, specifically expressed in skeletal muscle and located upstream of *MyoD* in the genome, can facilitate myogenesis by regulating *MyoD* expression [11]. *LncMyoD*, activated by MyoD, can regulate skeletal muscle differentiation by blocking IMP2-mediated mRNA translation [12]. *Linc-YY1* facilitates myogenic differentiation and muscle regeneration through its interaction with the transcription factor YY1 [13]. *Linc-RAM* promotes myogenic differentiation by directly interacting with MyoD [14]. LncRNA *SYISL* inhibits myogenesis by interacting with polycomb repressive complex 2 [15]. In addition, *linc-MD1* [16], *lnc-mg* [17], the lncRNA *MAR1* [18], the lncRNA *AK017368* [19] and the *Sirt1* AS lncRNA [20] control muscle differentiation and regeneration by functioning as competing endogenous RNAs (ceRNAs).

Natural antisense transcripts (NATs) are a group of a particular type of lncRNAs that are complementary to and overlap with the sense transcript of either protein-coding or non-protein-coding genes [21]. Based on the sharing of sequence segments between overlapping coding regions as well as the direction of transcription, the overlap pattern of natural antisense transcripts and sense transcripts can be divided into three categories [22]: 1) “head-to-head”, in which the sense and antisense transcripts overlap at their 5′ ends; 2) “tail-to-tail”, in which the sense and antisense transcripts overlap at their 3′ ends; and 3) “embedded overlap” (also called “full overlap”), in which one entire transcript overlaps the other [22]. NATs can regulate the expression of their sense gene pair or of several related genes, but the biological significance of this regulation remains under scientific investigation [23].

In the current study, we identified a novel natural antisense transcript derived from the opposite DNA strand of *Foxk1*, which partially overlaps the 3′ untranslated region (3′ UTR) of *Foxk1*, falling into the category of “embedded overlap”. This transcript was predicted to be a noncoding RNA and was named *Foxk1-AS*. We found that *Foxk1-AS* decreased the *Foxk1* expression level in vitro and in vivo and promoted myoblast differentiation and muscle regeneration. We further explored the regulatory mechanism of *Foxk1-AS* by analysing the expression of its downstream genes.

## Materials and methods

### Animals

Eight-week-old C57BL6 male wild-type (WT) mice were used. The animal procedures were performed according to protocols approved by the Animal Care and Use Committee of Tongji University School of Medicine (approval no. GB14924.2.). Maintenance conditions of animals (food: compound feed for experimental animals; temperature: 20–26 °C; humidity: 40–70/%). Each group contained 6 mice.

## C2C12 cell culture and differentiation

The murine myoblast cell line C2C12 was cultured in growth medium with high-glucose Dulbecco’s modified Eagle’s medium (DMEM) supplemented with 10% foetal bovine serum (FBS; Gibco, Grand Island, NY, USA) and 1% penicillin/streptomycin (15140,122; Gibco) in an incubator with 5% (v/v) CO_2_ at 37 °C. After the myoblasts reached 80%-90% confluence, the growth medium was removed and the cells were induced to differentiate into skeletal muscle cells by culture in Dulbecco’s modified Eagle’s medium supplemented with 2% horse serum (Gibco USA) for 6 days.

## Muscle injury and regeneration

Muscle injury induced by BaCl_2_ was performed as described below. In brief, 10 μL of 1.5% BaCl_2_ was injected intramuscularly into the left tibialis anterior (TA) muscle of eight-week-old C57BL/6 male WT mice. Phosphate-buffered saline (PBS) was injected into the right TA muscle of the same mice as the control. Muscle samples were then collected at the indicated days after injection, and regeneration and repair were assessed by haematoxylin–eosin (H&E) staining and qPCR. H&E staining of muscle sections was performed according to previous reports [17, 25], and images were acquired using an optical microscope (BX53; Olympus). MHC and MyoD expression in muscle samples was measured using quantitative real-time PCR (qRT–PCR).

## Overexpression and knockdown of Foxk1-AS in C2C12 cells and muscle tissues

To investigate the specific functions of Foxk1-AS, *Foxk1-AS* was overexpressed or knocked down in C2C12 cells using lentivirus infection and *Foxk1-AS* was overexpressed or knocked down in tibialis anterior muscle tissues using adeno-associated virus infection. *Foxk1-AS* was cloned into the lentiviral vector GV303-GFP (GeneChem, Shanghai, China) for overexpression in C2C12 cells, and empty plasmid without an insertion sequence was used as the negative control. The siRNA sequences of *Foxk1-AS* (siRNA-Foxk1-AS 1: CCACATACAACAACCGAAT; siRNA-Foxk1-AS 2: TAGCCCAAGTACAGAAACA; siRNA-Foxk1-AS 3: ACACAAGGACTTAGGGGAA) were cloned into the lentiviral vector GV118-GFP (GeneChem, Shanghai, China) to inhibit the expression of *Foxk1-AS* in C2C12 cells, and an siRNA without sequence homology to mouse genes was used as the negative control. The full length *Foxk1-AS* or siRNA-Foxk1-AS 1 sequence of *Foxk1-AS* was cloned into the adeno-associated viral vector GV478 (GeneChem, Shanghai, China) to achieve overexpression or knockdown of *Foxk1-AS* in muscle tissues. Viral infection of cells and tissues was performed according to the manufacturer’s instructions.

## Isolation of RNA and quantitative real-time PCR

Total RNA was extracted from tissues or cells using TRIzol reagent (Invitrogen Life Technologies, Carlsbad, CA, USA) according to the manufacturer’s instructions, and a PrimeScript RT Reagent Kit (TaKaRa, Otsu, Japan) was used for cDNA synthesis. Quantitative real-time PCR was performed using SYBR Green PCR Master Mix. The primers specific for the target genes were as follows: Foxk1-AS (F: ACTCGGATTCCCATCGTATTGG; R: CTCTTCCCCTAAGTCCTTGTGT); Foxk1 (F: AAGCATTCCGAAAGCGGAGA; R: CTGGAACGAGGGGACATCAG); MHC (F: CGCAAGAATGTTCTCAGGCT; R: GCCAGGTTGACATTGGATTG); MyoG (F: GTCCCAACCCAGGAGATCAT; R: CCACGATGGACGTAAGGGAG); MyoD (F: CATCCGCTACATCGAAGGTC; R: GTGGAGATGCGCTCCACTAT); Mef2c (F: CATAACATGCCGCCATCTGC; R: CGCTCCCATCGTAGGAACTG); Myoz1 (F: GGGGGTTGATCCTCAGCAAA; R: TGGAAGGTCATGCGTTTGGA); Clcn1 (F: TGGCCTCCATCTTGCCTATG; R: TTGTATTCCGGAAGCTGGCA); Myl1 (F: TTGAGGGTCTGCGTGTCTTC; R: GATGCAGCCATTGGAGTCCT); Pfkm (F: GGATCTTTGCCAACACCCCT; R: GAGGATTGGCCTCAGCTTCA); Pgam2 (F: CCTTCGGGGCATTGTGAAAC; R: TCGTCTCCCAGGAACCTCAT); Ryr1 (F: ACCCCACATGGGTTTGAGAC; R: GGGAAGAAATCCCAGCACCT); Ttn (F: AATGCTCAAAGCAGGCGGAA; R: TCCTTCCACCACAGGACCAT); mGAPDH (F: CAGGTTGTCTCCTGCGACTT; R: CCCTGTTGCTGTAGCCGTAT).

## Nuclear–cytoplasmic fractionation

C2C12 cells were harvested and lysed in lysis buffer containing ribonucleoside–vanadyl complex (10 mM; New England Biolabs) and protease inhibitor cocktail (Calbiochem). After a brief centrifugation procedure, the pellet was preserved as the nuclear fraction. The supernatant was preserved as the cytoplasmic fraction.

## Western blotting

C2C12 cells were infected with *Foxk1-AS* overexpression lentivirus, and tibialis anterior muscle tissues were infected with *Foxk1-AS* overexpression adeno-associated virus. Cells and muscle tissues were collected and lysed in RIPA lysis buffer. Western blotting was performed using a standard procedure. The primary antibodies used were specific for MyoD (554130; BD), MHC (MAB4470; R&D), β-tubulin (66240-1-Ig; Proteintech), GAPDH (60004-1-Ig; Proteintech) and Foxk1(ab18196; Abcam).

## Immunofluorescence

C2C12 cells were infected with *Foxk1-AS* overexpression lentivirus and control virus. Twenty-four hours after infection, skeletal muscle differentiation was induced in C2C12 cells for 3 days, and immunofluorescence staining was performed. Myotubes were stained with an anti-MHC antibody (Millipore, #05-833), and the nuclei were stained with 4', 6-diamidino-2-phenylindole (DAPI). An anti-mouse secondary antibody was used (Invitrogen). Images were acquired with a fluorescence microscope (CX41-32RFL, Olympus, Japan).

## Statistical analysis

Statistical analyses were carried out using Student’s t test. All data are presented as the mean standard deviation (s.d.) values. Probability (P) values of less than 0.05 were considered to be statistically significant.

## Results

### Identification of *Foxk1-AS*

**Fig. 1:**
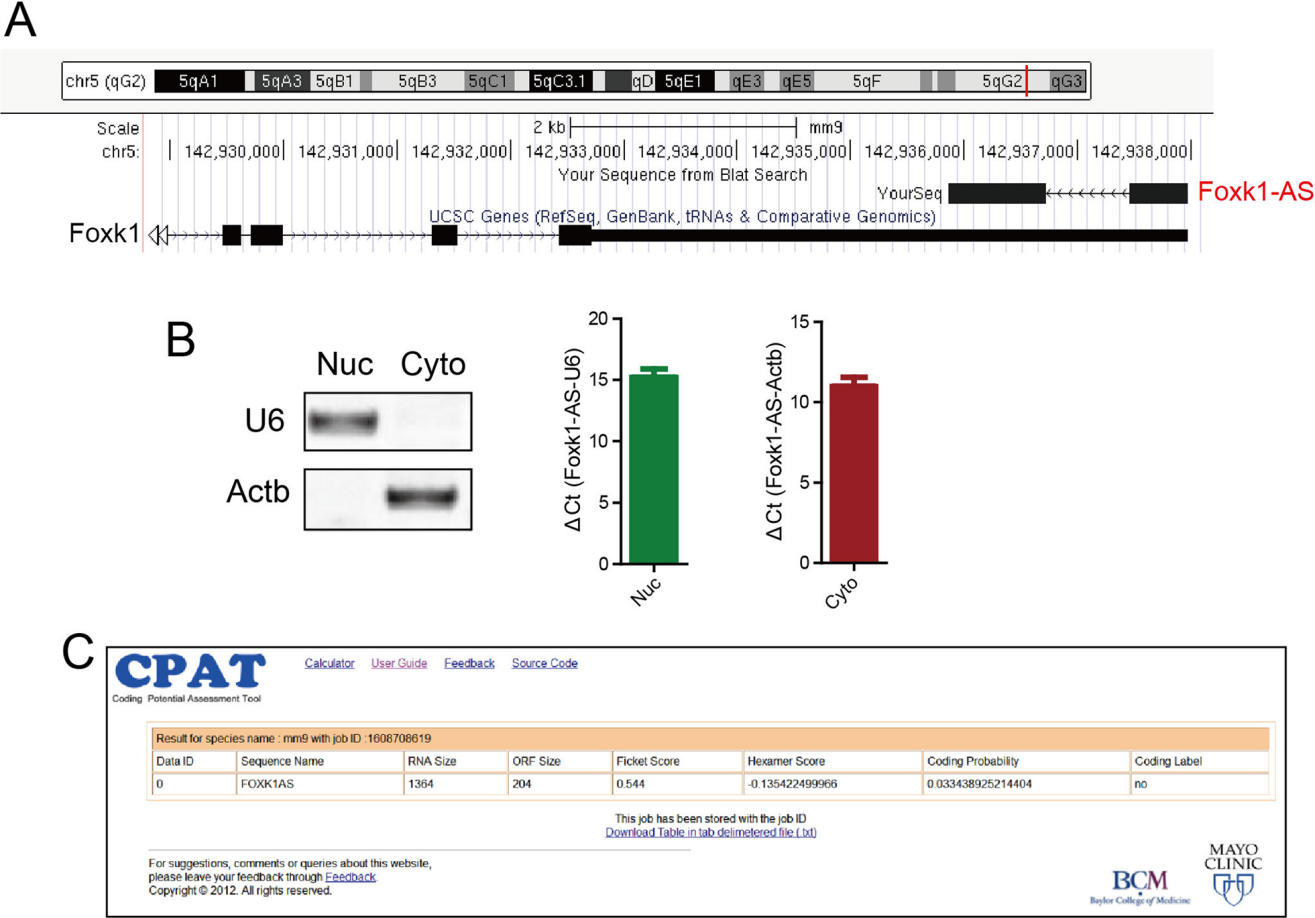
Identification of Foxk1-AS. **A** Foxk1-AS is located on chromosome 5 in mice. Foxk1-AS is transcribed from the opposite DNA strand of Foxk1 and completely incorporated in the 3′ UTR of Foxk1. **B** Foxk1-AS was enriched in the cytoplasm and nucleus. **C** Foxk1-AS was predicted to be a noncoding RNA

To date, tens of thousands of novel long noncoding RNAs (lncRNAs) have been identified by using RNA-seq; however, their functions remain largely unexamined. We previously identified more than 600 long noncoding RNAs (lncRNAs) by single cell transcriptome analysis [24], and here, we focused our study on antisense lncRNAs because most of them could function as regulators of sense genes. We found a novel natural antisense RNA, which is transcribed from the opposite DNA strand of *Foxk1* and therefore named *Foxk1-AS*. *Foxk1-AS*, not annotated previously, contains two exons and is completely incorporated into the 3′ UTR of *Foxk1* (Fig. 1A). Cell fractionation followed by qRT–PCR demonstrated that *Foxk1-AS* resides both in the nucleus and cytoplasm (Fig. 1B). The Coding Potential Assessment Tool (CPAT) computational algorithm predicted that *Foxk1-AS* contains 1364 nucleotides (nt) (Additional file 1: Fig. S1A) and has a very low coding potential (Fig. 1C); thus, *Foxk1-AS* was predicted to be noncoding RNA.

## The expression patterns of *Foxk1* and *Foxk1-AS* during myoblast differentiation

**Fig. 2:**
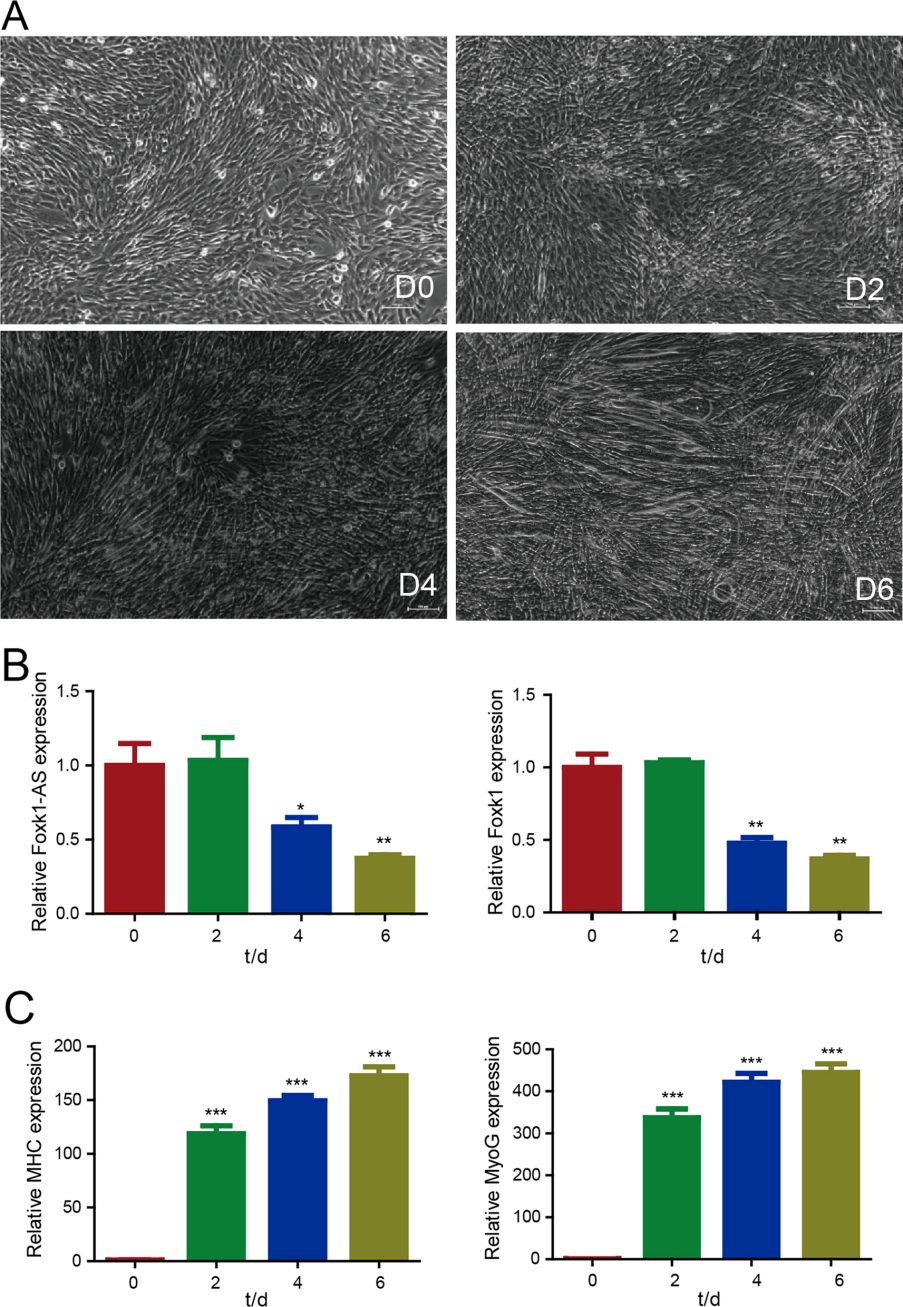
The expression patterns of Foxk1-AS, Foxk1, MHC and MyoG during myoblast differentiation. **A** Myogenic differentiation of C2C12 cells on Day 0, Day 2, Day 4 and Day 6. **B** Relative expression of Foxk1-AS, Foxk1, MHC and MyoG during myoblast differentiation on Day 0, Day 2, Day 4 and Day 6

To investigate the role of Foak1-AS in myogenesis, we examined the expression pattern of Foxk1-AS, Foxk1, MyoG and Myosin Heavy Chain (MHC), a marker of terminal muscle differentiation during myogenic differentiation. We cultured mouse skeletal muscle C2C12 cells in high (10%) foetal calf serum medium (which has been used as a model of undifferentiated myoblasts) and then switched them to low (2%) horse serum medium to initiate myogenic differentiation (which has been used as a model of differentiated myoblasts) (Fig. 2A). RNA was extracted from undifferentiated and differentiated myoblasts, and the gene expression patterns of *Foxk1-AS*, *Foxk1* and myogenic differentiation-related genes were examined by RT–PCR. The expression levels of Foxk1-AS and Foxk1 were decreased in C2C12 cells from Day 4 to Day 6 of differentiation (Fig. 2B), whereas those of MyoG and MHC increased gradually (Fig. 2C) from Day 2 to Day 6 during the early differentiation of myoblasts into myotubes. These findings suggested that Foxk1-AS and Foxk1 have a functional role in the myogenic differentiation of C2C12 cells.

## *Foxk1-AS* promoted myoblast differentiation by reducing the expression of *Foxk1* in C2C12 cells

**Fig. 3:**
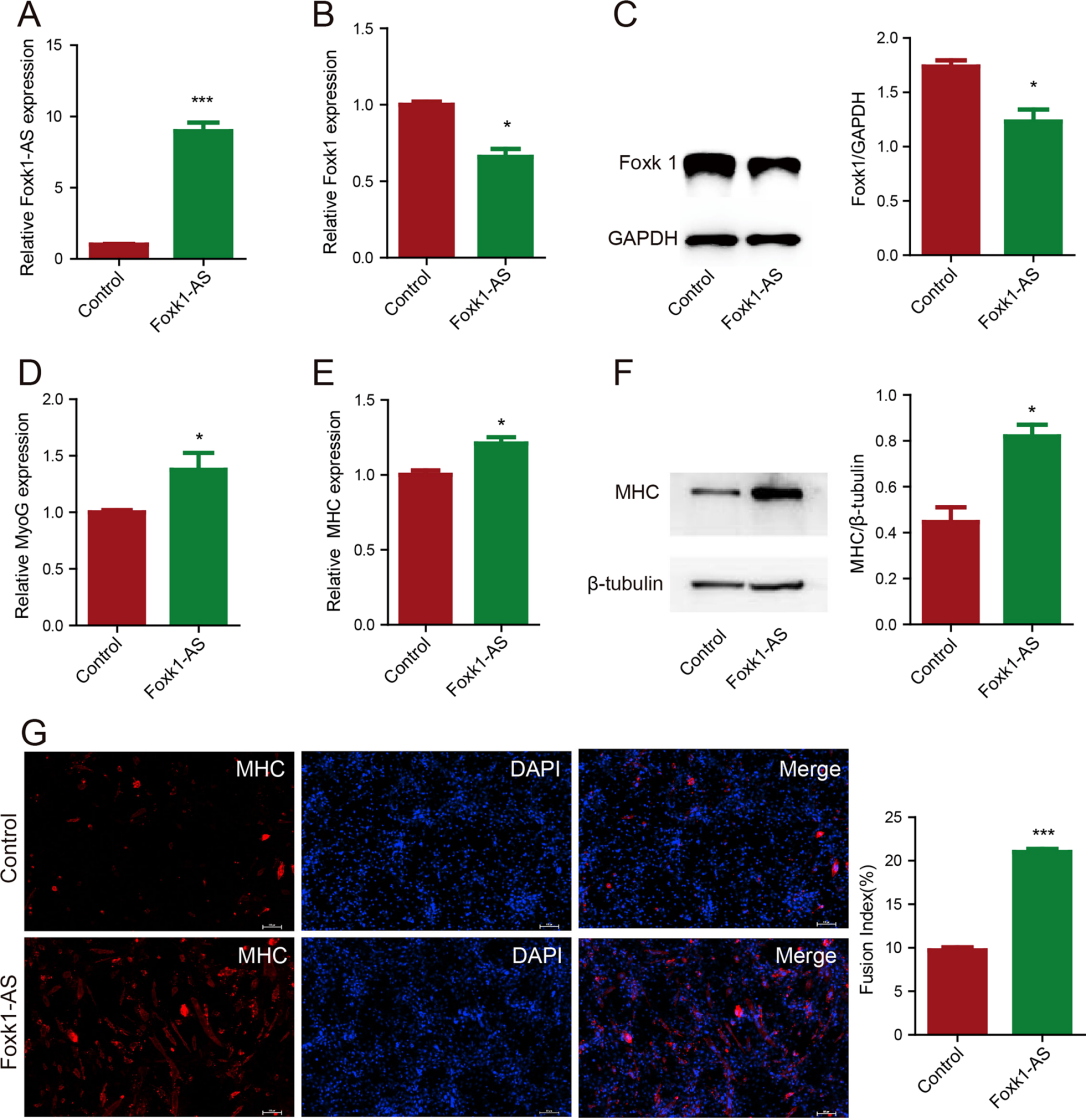
Foxk1-AS promoted myoblast differentiation by reducing the expression of Foxk1 in C2C12 cells. (**A**)(**B**) Overexpression of Foxk1-AS decreased the expression of the Foxk1 gene at the transcriptional level in C2C12 cells. (**C**) Overexpression of Foxk1-AS decreased the expression of the Foxk1 gene at the translational level in C2C12 cells. (**D**) Overexpression of Foxk1-AS increased the expression of the MyoG gene at the transcriptional level in C2C12 cells. (**E**) Overexpression of Foxk1-AS increased the expression of the MHC gene at the transcriptional level in C2C12 cells. (**F**) Overexpression of Foxk1-AS increased the expression of the MHC gene at the translational level in C2C12 cells. (**G**) Overexpression of Foxk1-AS increased MHC staining on Day 3 after skeletal muscle differentiation (left), and the fusion index was calculated (right). The fusion index was defined as the ratio of the number of nuclei in myotubes to the total number of nuclei

To investigate the regulatory effect of Foxk1-AS on *Foxk1* expression, we constructed *Foxk1-AS* overexpression and knockdown lentiviruses and used them to infect C2C12 cells. The expression level of Foxk1-AS in C2C12 cells infected with *Foxk1-AS* overexpression lentivirus was successfully increased by approximately tenfold (Fig. 3A), and in the knockdown experiments, two of the three knockdown lentiviruses successfully decreased the Foxk1-AS level by 40% (Additional file 1: Fig. S2A). Overexpression of *Foxk1-AS* in C2C12 cells downregulated the expression of Foxk1 mRNA and protein (Fig. 3B, 3C), suggesting that Foxk1 is a target negatively regulated by Foxk1-AS. However, knockdown *Foxk1-AS* had no impact on *Foxk1* gene expression (Additional file 1 : Fig. S2B), possibly due to the low basal expression level of Foxk1-AS in C2C12 cells; therefore, knockdown of *Foxk1-AS* was not further pursued.

Previous research showed that Foxk1 inhibits myogenic differentiation [7]. This study showed that Foxk1-AS represses *Foxk1* gene expression. To examine the effect of *Foxk1-AS* on myogenic differentiation, we overexpressed *Foxk1-AS* in C2C12 cells and then induced them to undergo myogenic differentiation. We found that overexpression of *Foxk1-AS* resulted in augmented terminal differentiation, as demonstrated by upregulated levels of MyoG mRNA (Fig. 3D), upregulated the levels of MHC mRNA and protein (Fig. 3E, 3F) and increased MHC staining (Fig. 3G). Collectively, these studies support the model—that is, Foxk1-AS promotes myoblast differentiation by reducing the expression of *Foxk1* in C2C12 cells.

## Foxk1-AS downregulated the expression of *Foxk1* in skeletal muscle tissue

**Fig. 4:**
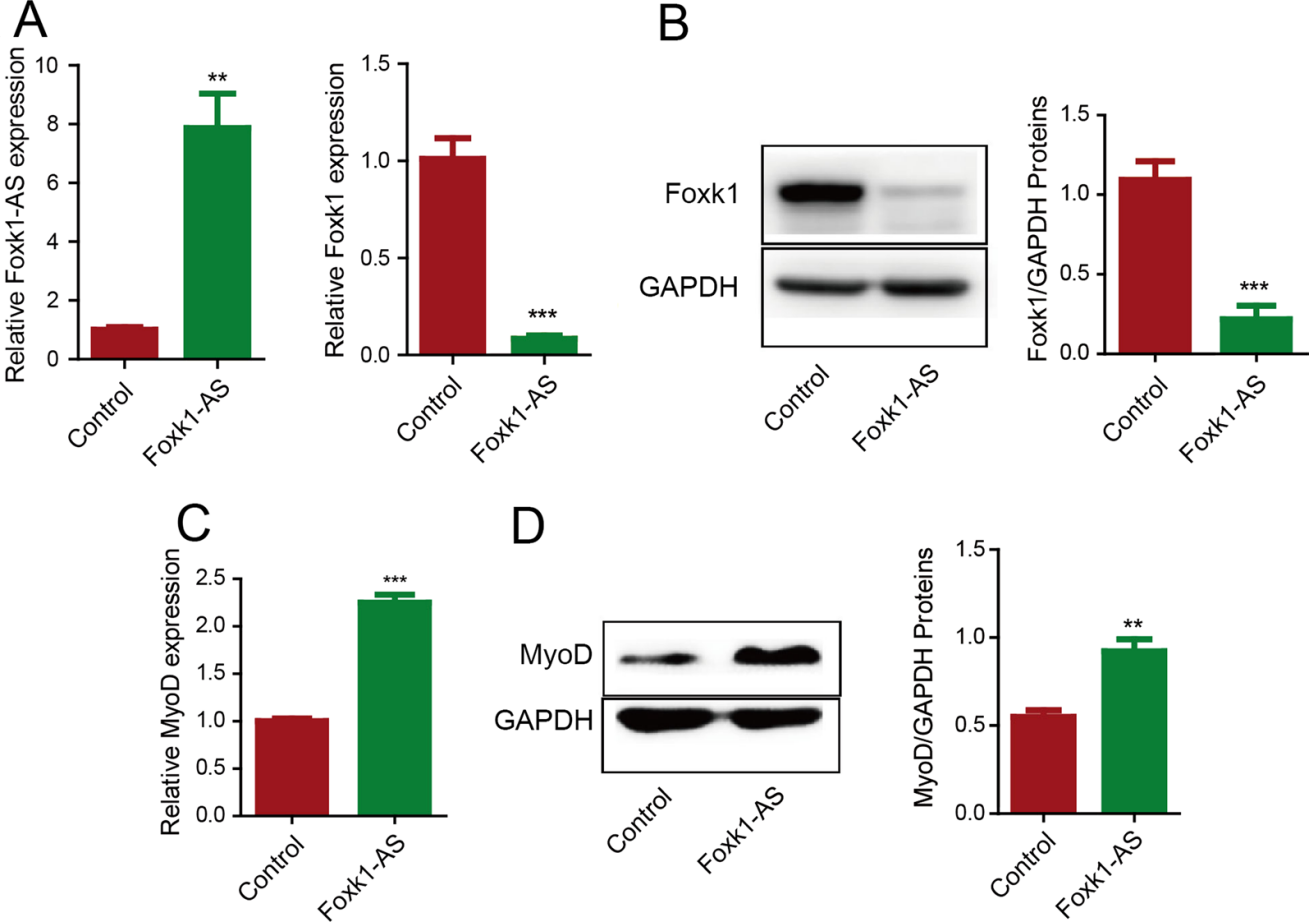
Foxk1-AS downregulated the expression of Foxk1 in skeletal muscle tissue. **A** Overexpression of Foxk1-AS decreased the expression of the Foxk1 gene at the transcriptional level in the tibialis anterior. **B** Overexpression of Foxk1-AS decreased the expression of the Foxk1 gene at the translational level in the tibialis anterior. **C** Overexpression of Foxk1-AS increased the expression of the MyoD gene at the transcriptional level in the tibialis anterior. **D** Overexpression of Foxk1-AS increased the expression of the MyoD gene at the translational level in the tibialis anterior

To further confirm the regulatory effect of Foxk1-AS on *Foxk1* expression in vivo, we constructed *Foxk1-AS* overexpression and knockdown adenoviruses and injected them into the tibialis anterior muscle. Consistent with the in vitro results, knockdown of Foxk1-AS had no effect on the gene expression of *Foxk1* (Figure S2C) due to the low basal expression level of *Foxk1-AS* in the tibialis anterior. Overexpression of *Foxk1-AS* in the tibialis anterior downregulated the levels of Foxk1 mRNA and protein (Fig. 4A, 4B), and increased the expression level of MyoD, one of the most critical transcription factors, which regulates myogenesis and is only expressed in activated myosatellite cells (Fig. 4C, 4D). These results further confirmed the role of Foxk1-AS in reducing the expression of *Foxk1* in skeletal muscle tissue and thus promoting myogenesis.

## Foxk1-AS promotes the regeneration of damaged muscle fibres

**Fig. 5:**
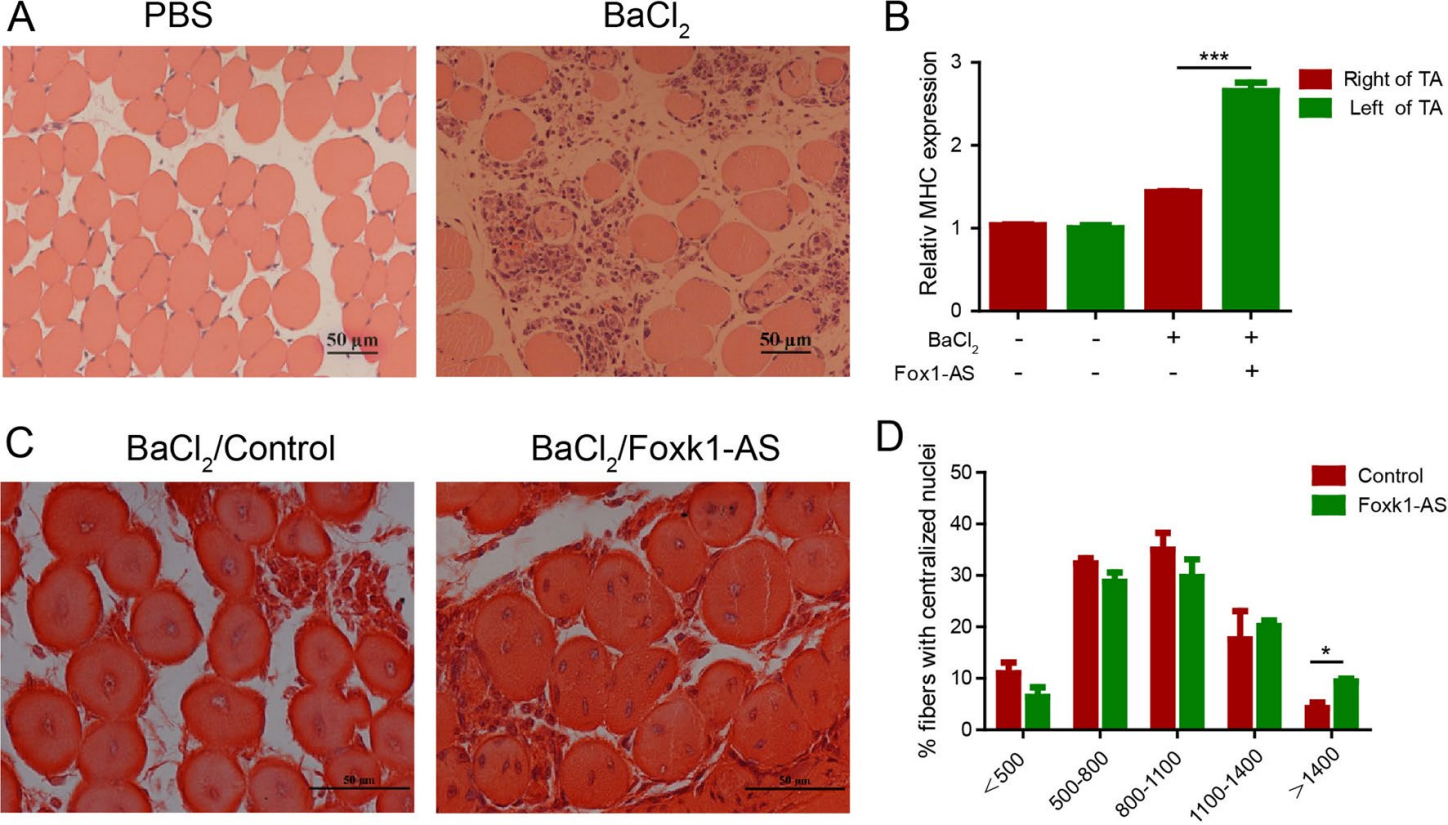
Foxk1-AS promotes the regeneration of damaged muscle fibres. **A** BaCl_2_ can cause different degrees of damage to skeletal muscle fibers. **B** Overexpression of Foxk1-AS upregulated the levels of MHC mRNA on Day 3 after injection of BaCl_2_. **C** The number of multinucleated myotubes was much higher in the Foxk1-AS overexpression group than in the control group at 7 days after injection of BaCl_2_. **D** The proportion of regenerated muscle fibres with a central nucleus of more than 1400 square microns in the Foxk1-AS overexpression group was significantly higher than that in the control group

To explore whether Foxk1-AS is implicated in the regulation of damaged muscle fibre regeneration, we injected BaCl_2,_ which can cause different degrees of damage to skeletal muscle fibres, into the tibialis anterior (TA) muscle of mice 3 days after injection of *Foxk1-AS* overexpression adenovirus (Fig. 5A). Then, we analysed the expression of myosin heavy chain (MHC), the basic unit of fibromyosin in skeletal muscle, on Day 3 after injection of BaCl_2_. We found that *MHC* expression in the BaCl_2_ group was higher than that in the untreated group because when skeletal muscle is damaged by BaCl_2_, the myosin structure is destroyed, which stimulates the expression of *MHC* genes to repair damaged muscle fibres. During regeneration, overexpression of *Foxk1-AS* upregulated the levels of MHC mRNA (Fig. 5B) compared with the control, suggesting that Foxk1-AS regulates muscle satellite cells and muscle stem cells and promotes the regeneration of damaged skeletal muscle in mice. In support of this notion, we observed that at 7 days after injection of BaCl_2_, the number of multinucleated myotubes was much higher in the Foxk1-AS overexpression group than in the control group (Fig. 5C). In addition, the proportion of regenerated muscle fibres with a central nucleus of more than 1400 square microns in the Foxk1-AS overexpression group was significantly higher than that in the control group (Fig. 5D). Together, the above results convincingly reveal the function of Foxk1-AS in promoting myogenic differentiation during muscle development and the regeneration of damaged muscle fibres.

## Foxk1-AS rescues Mef2c activity by repressing Foxk1 expression and thereby promoting the regeneration of damaged muscle fibres

**Fig. 6:**
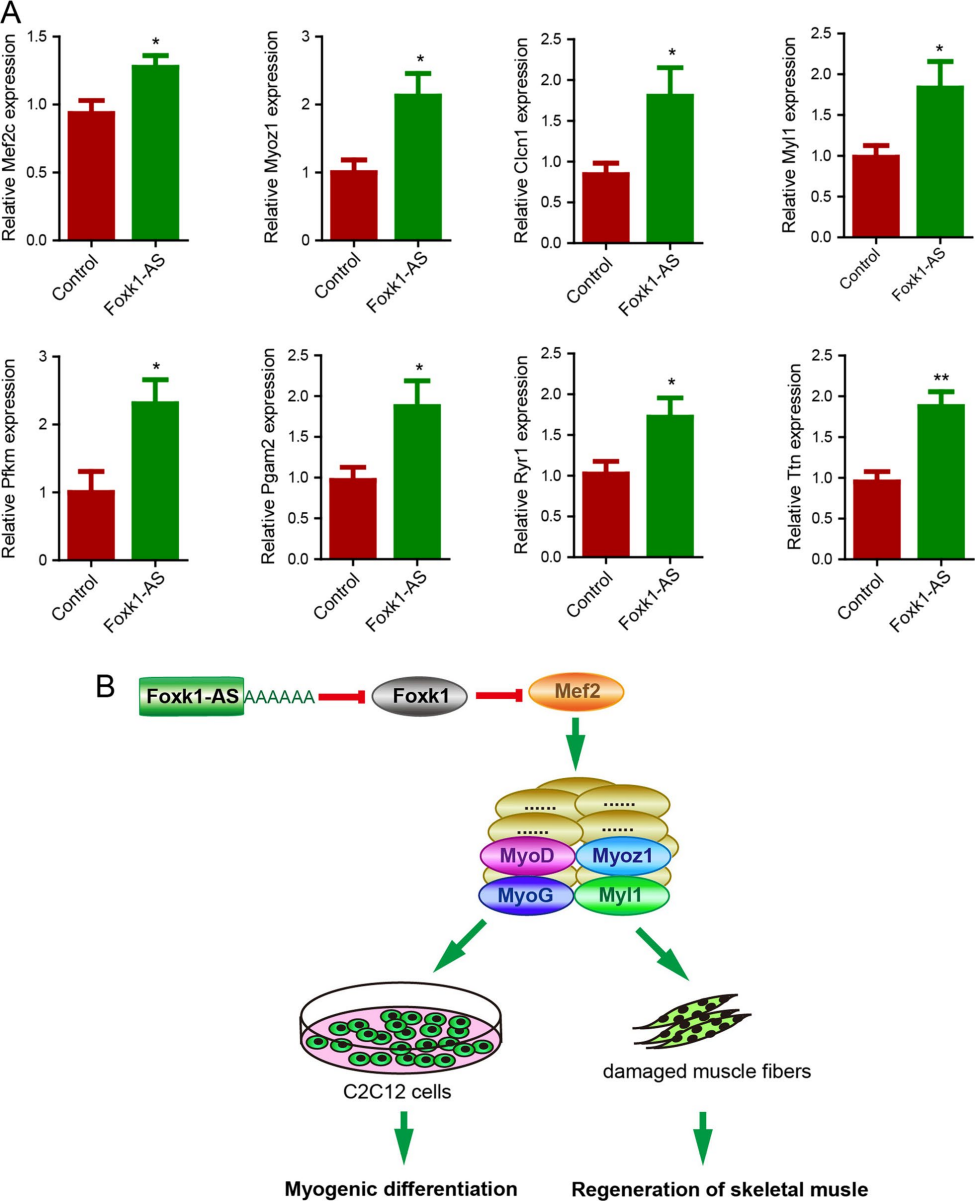
Foxk1-AS represses Foxk1, thereby rescuing Mef2c activity and promoting the regeneration of damaged muscle fibres. **A** Overexpression of Foxk1-AS in damaged muscle fibres led to an increase in the mRNA levels of Mef2c and its target genes. **B** Foxk1-AS represses Foxk1, thereby rescuing Mef2c activity and promoting the regeneration of damaged muscle fibres

Myocyte enhancer factor 2 (Mef2) is a conserved and important transcription factor that controls muscle gene expression. Mef2 acts as a transcription activator by binding specifically to Mef2 elements present in the regulatory regions of several muscle-specific genes [3]. There are four closely related *Mef2* genes: *Mef2a, -b, -c* and *-d* [3]. Mef2c has both DNA binding and transactivating activities and plays a role in maintaining the differentiated state of muscle cells. Previous research demonstrated that Foxk1 binds to *Mef2c* and represses its transcriptional activity, thereby inhibiting myogenic differentiation [7]. We analysed the expression of *Mef2c* and some Mef2 target genes, including myozenin 1 (*Myoz1*), chloride voltage-gated channel 1 (*Clcn1*), myosin light chain 1 (*Myl1*), phosphofructokinase muscle (*Pfkm*), phosphoglycerate mutase 2 (*Pgam2*), ryanodine receptor 1 (*Ryr1*) and titin (*Ttn*), in damaged tibialis anterior muscle with or without overexpression of *Foxk1-AS* and found that the expression levels of *Mef2c* and Mef2 target genes were elevated in damaged tibialis anterior muscle with overexpression of *Foxk1-AS* (Fig. 6A). Mechanistically, Foxk1-AS downregulates the expression of Foxk1, and Foxk1 binds to Mef2c and represses its transcriptional activity. These studies support the idea that Foxk1-AS downregulates the expression of *Foxk1*, thereby rescuing Mef2c activity and promoting the regeneration of damaged muscle fibres (Fig. 6B).

## Discussion

NATs are transcribed from the opposite strand of a coding or noncoding gene and are relatively less studied than other RNAs. NATs can regulate specific gene expression and thus may be regarded as a potential target for gene therapy for diseases associated with dysregulation of certain genes; however, only a small number of NATs have been functionally studied. In this paper, we report the identification of a novel NAT, Foxk1-AS, which targets *Foxk1* and functions as a regulator of myogenesis. Foxk1-AS negatively regulates the expression of *Foxk1* in C2C12 cells and in the tibialis anterior and promotes myoblast differentiation and the regeneration of muscle fibres damaged by BaCl_2_.

Embedded overlap is one category of NAT overlap patterns; in this pattern, the entire natural antisense transcript overlaps the sense transcript and may originate from any part of a given protein-coding gene. Previous research has shown that Sirt1 AS overlaps the 3′ UTR of *Sirt1* and interacts with Sirt1 mRNA to form an RNA duplex that promotes Sirt1 translation by competing with miR-34a, thereby, inhibiting muscle formation [25]. Similar to Sirt1 AS, Foxk1-AS overlaps the 3′ UTR of *Foxk1*, but it reduces rather than promotes the expression of the sense transcript *Foxk1*, suggesting that although the structures are the same, the functional roles of NATs are different. The functional roles of NATs in regulating their sense genes could include activation, suppression or homeostatic modulation, depending on the mechanism underlying the function.

Different subcellular localizations of NATs lead to different biological functions. NATs in the cytoplasm mainly regulate RNA stability and/or mRNA translatability. However, NATs accumulated in the nucleus are mainly involved in alternative splicing and RNA processing [23, 26]. The antisense transcripts of phosphatase and tensin homologue (PTEN) [27], tumour protein p73 (TP73) [28], G protein subunit gamma 12 (GNG12) [29] and eosinophil granule ontogeny transcript (EGOT) [30] accumulate in the nucleus and may play potential roles in mediating gene expression via epigenetic modification, transcriptional interference and alternative splicing. In contrast, the cytoplasmic antisense transcripts of SRY-box transcription factor 9 (SOX9) [31], rhophilin rho GTPase binding protein 1 (RHPN1) [32], forkhead box D 2 (FOXD2) [33], and homeobox D (HOXD) [34] can modulate the expression of their sense transcripts or other genes by acting as miRNA sponges. Some NATs, such as the antisense transcripts of homeobox A11 (HOXA11) [35, 36] and zinc finger E-box binding homeobox 1 (ZEB1) [37, 38], accumulate in both the nucleus and cytoplasm and can participate in multiple processes of gene expression through epigenetic modification and miRNA ponging mechanisms. Unlike the cytoplasmic Sirt1 AS, which overlaps the 3′ UTR of *Sirt1*, Foxk1-AS accumulates in both the nucleus and cytoplasm, and the different biological functions between Foxk1-AS and Sirt1 AS may be ascribed to their different subcellular localization.

## Conclusion

In summary, our study indicated that Foxk1-AS represses *Foxk1*, thereby rescuing Mef2c activity and promoting myogenic differentiation of C2C12 cells and the regeneration of damaged muscle fibres. Our study provides new insight into the molecular mechanisms by which NATs induce the regeneration of damaged muscle fibres, as well as a new therapeutic strategy for repairing damaged muscle fibres.

## Supplementary Information

**Additional file 1: Figure S1.** The sequence of Foxk1-AS. The boundary of exon and intron is marked in red. **Figure S2**. Knockdown of Foxk1-AS in C2C12 cells and tibialis anterior have no effect on gene expression of Foxk1. (**A**) Two of three knockdown lentivirus successfully decreased Foxk1-AS levels by 40% in C2C12 cells. (**B**) Knockdown of Foxk1-AS in C2C12 cells have no effect on gene expression of Foxk1. (**C**) Knockdown of Foxk1-AS in tibialis anterior have no effect on gene expression of Foxk1.

## Data Availability

All data in our study are available upon request.

## Abbreviations

NATs: Natural antisense transcripts
3′ UTR: 3′ Untranslated region
PBS: Phosphate-buffered saline
WT: Wild-type
TA: Tibialis anterior
FBS: Foetal bovine serum
H&E: Haematoxylin–eosin
CPAT: Coding potential assessment tool
MHC: Myosin Heavy Chain
qRT–PCR: Quantitative real-time PCR
MyoG: Myogenic regulatory factors myogenin
MyoD: Myogenic differentiation 1
Myf5: Myogenic factor 5
Mef2: Myocyte enhancer factor 2
