# Supplementary material for: Natural antisense RNA Foxk1-AS promotes myogenic differentiation by inhibiting Foxk1 activity
Source: Cell Commun Signal. 2022 May 31;20:77. doi: 10.1186/s12964-022-00896-2 (PMC9158385; doi:10.1186/s12964-022-00896-2)
Supplement: Supplementary file 2 — Additional file 1: Figure S1. The sequence of Foxk1-AS. The boundary of exon and intron is marked in red. Figure S2. Knockdown of Foxk1-AS in C2C12 cells and tibialis anterior have no effect on gene expression of Foxk1. (A) Two of three knockdown lentivirus successfully decreased Foxk1-AS levels by 40% in C2C12 cells. (B) Knockdown of Foxk1-AS in C2C12 cells have no effect on gene expression of Foxk1. (C) Knockdown of Foxk1-AS in tibialis anterior have no effect on gene expression of Foxk1. [file 12964_2022_896_MOESM2_ESM.ppt]

## Slide 1
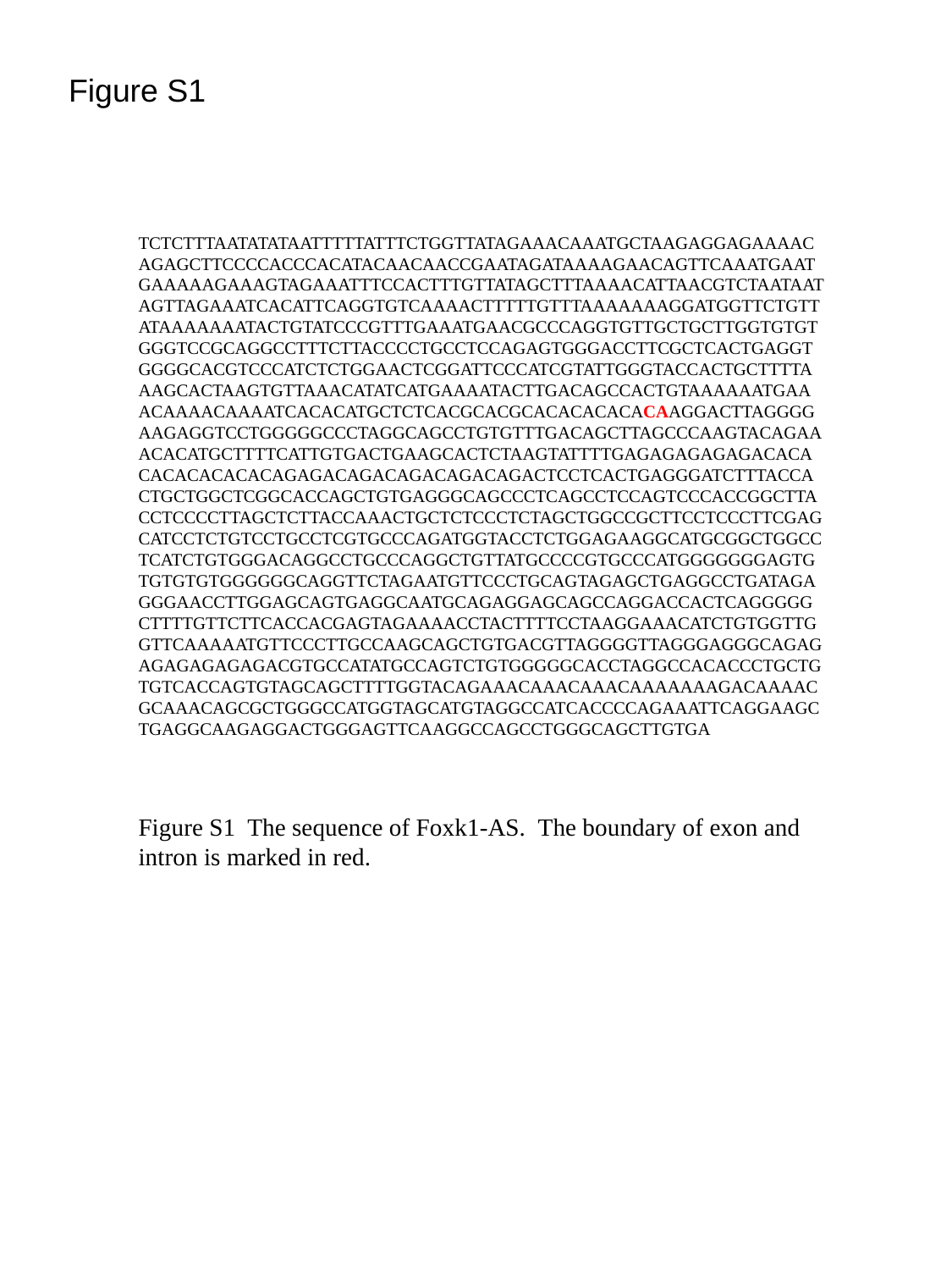

Figure S1
TCTCTTTAATATATAATTTTTATTTCTGGTTATAGAAACAAATGCTAAGAGGAGAAAACAGAGCTTCCCCACCCACATACAACAACCGAATAGATAAAAGAACAGTTCAAATGAATGAAAAAGAAAGTAGAAATTTCCACTTTGTTATAGCTTTAAAACATTAACGTCTAATAATAGTTAGAAATCACATTCAGGTGTCAAAACTTTTTGTTTAAAAAAAGGATGGTTCTGTTATAAAAAAATACTGTATCCCGTTTGAAATGAACGCCCAGGTGTTGCTGCTTGGTGTGTGGGTCCGCAGGCCTTTCTTACCCCTGCCTCCAGAGTGGGACCTTCGCTCACTGAGGTGGGGCACGTCCCATCTCTGGAACTCGGATTCCCATCGTATTGGGTACCACTGCTTTTAAAGCACTAAGTGTTAAACATATCATGAAAATACTTGACAGCCACTGTAAAAAATGAAACAAAACAAAATCACACATGCTCTCACGCACGCACACACACACAAGGACTTAGGGGAAGAGGTCCTGGGGGCCCTAGGCAGCCTGTGTTTGACAGCTTAGCCCAAGTACAGAAACACATGCTTTTCATTGTGACTGAAGCACTCTAAGTATTTTGAGAGAGAGAGACACACACACACACACAGAGACAGACAGACAGACAGACTCCTCACTGAGGGATCTTTACCACTGCTGGCTCGGCACCAGCTGTGAGGGCAGCCCTCAGCCTCCAGTCCCACCGGCTTACCTCCCCTTAGCTCTTACCAAACTGCTCTCCCTCTAGCTGGCCGCTTCCTCCCTTCGAGCATCCTCTGTCCTGCCTCGTGCCCAGATGGTACCTCTGGAGAAGGCATGCGGCTGGCCTCATCTGTGGGACAGGCCTGCCCAGGCTGTTATGCCCCGTGCCCATGGGGGGGAGTGTGTGTGTGGGGGGCAGGTTCTAGAATGTTCCCTGCAGTAGAGCTGAGGCCTGATAGAGGGAACCTTGGAGCAGTGAGGCAATGCAGAGGAGCAGCCAGGACCACTCAGGGGGCTTTTGTTCTTCACCACGAGTAGAAAACCTACTTTTCCTAAGGAAACATCTGTGGTTGGTTCAAAAATGTTCCCTTGCCAAGCAGCTGTGACGTTAGGGGTTAGGGAGGGCAGAGAGAGAGAGAGACGTGCCATATGCCAGTCTGTGGGGGCACCTAGGCCACACCCTGCTGTGTCACCAGTGTAGCAGCTTTTGGTACAGAAACAAACAAACAAAAAAAGACAAAACGCAAACAGCGCTGGGCCATGGTAGCATGTAGGCCATCACCCCAGAAATTCAGGAAGCTGAGGCAAGAGGACTGGGAGTTCAAGGCCAGCCTGGGCAGCTTGTGA
Figure S1 The sequence of Foxk1-AS. The boundary of exon and intron is marked in red.

## Slide 2
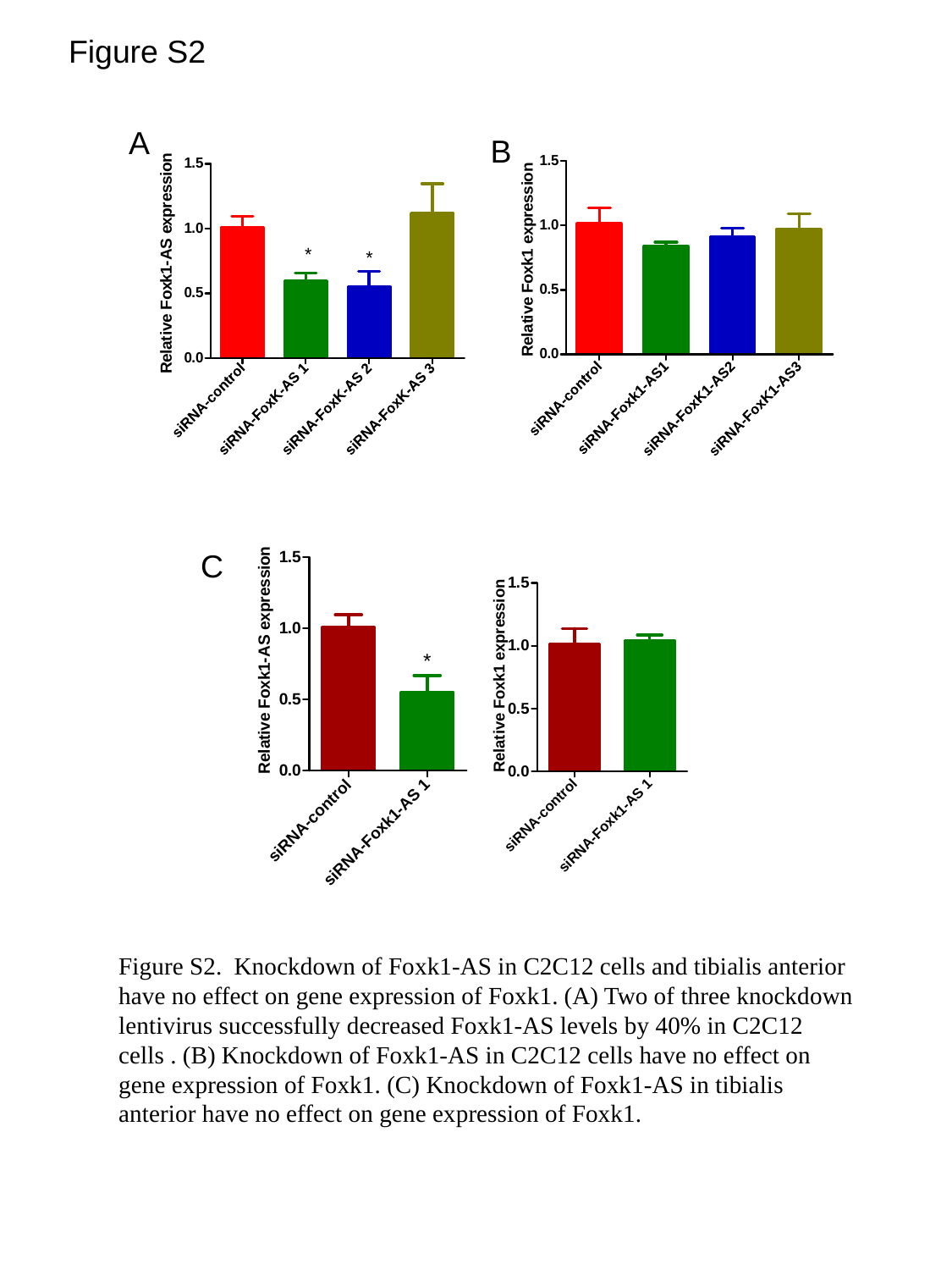

Figure S2
A
B
C
Figure S2. Knockdown of Foxk1-AS in C2C12 cells and tibialis anterior have no effect on gene expression of Foxk1. (A) Two of three knockdown lentivirus successfully decreased Foxk1-AS levels by 40% in C2C12 cells . (B) Knockdown of Foxk1-AS in C2C12 cells have no effect on gene expression of Foxk1. (C) Knockdown of Foxk1-AS in tibialis anterior have no effect on gene expression of Foxk1.
